# Supplementary material for: Are carbon emissions trading and green financial instruments synergistic? -Comprehensive quantitative research based on content analysis
Source: PLoS One. 2024 Mar 7;19(3):e0298601. doi: 10.1371/journal.pone.0298601 (PMC10919601; doi:10.1371/journal.pone.0298601)
Supplement: S1 Data — (ZIP) [file pone.0298601.s001.zip › Data/地级市Z_F工作报告.zip!/║┌┴·╜¡/║╫╕┌╩╨/└┤╫╘╣1⁄2╓┌║┼í╛┬φ┐╦╩2╛▌═°í┐_2019.docx]

各位代表：

现在，我代表市政府向大会作政府工作报告，请予审议，并请各位政协委员和其他列席的同志提出意见。

一、2018年工作回顾

2018年，是鹤岗推动城市转型发展、全面建成小康社会取得新进展的一年。面对复杂多变的形势、经济下行的压力和繁重紧迫的任务，特别是受地方煤矿整治整合、部分企业效益下滑、市区财税收入减缓、地方政府性债务化解的冲击，我们始终以党的十九大精神和习近平总书记系列重要讲话精神为指导，在省委、省政府和市委的坚强领导下，牢牢把握大局，团结一致，攻坚克难，经济社会在困境中实现了持续稳步发展。

据初步统计，2018年全市地区生产总值同比增长5%；规模以上工业增加值增长7%；固定资产投资增长4%；公共财政收入增长9.1%；社会消费品零售总额增长8%；外贸进出口总值下降30%；城镇常住居民人均可支配收入增长6.5%，农村常住居民人均可支配收入增长7%。

——过去的一年，我们着力破解制约全面建成小康社会的重点难点问题，强责任、抓落实，扎实推进“三大攻坚战”。有效防控金融风险。加强地方政府性债务管控，化解债务30亿元。扎实推进精准脱贫。60个贫困村全部实现“三通三有”，“两不愁三保障”各项政策全面落实。绥滨县代表我省接受国家第三方评估考核、国务院督查。坚决防治生态环境污染。加强地表水饮用水水源保护工作，落实中央环保督察“回头看”意见整改，严禁秸秆露天焚烧，拆除燃煤小锅炉279台、整治油烟污染企业326户。强力推进违法用地专项行动，处置违法用地400公顷。以创新和为民担当的情怀全面推进“两河十四沟”清水秀岸黑臭水体综治工程，一期整治7条河沟、19公里。我市成为第一批全国水生态文明城市。

——过去的一年，我们牢牢把握稳中求进的工作总基调，转方式、调结构，经济发展质量逐步提高。生态农业加快发展。粮食综合生产能力巩固提升，粮食产量预计达到20亿斤。畜牧业稳步提升。工业经济企稳增效。全市规模以上工业企业预计新增26户，用电量同比增长8.5%。帮助29户规上停产企业恢复生产、亏损面减少5.6%。落实结构性减税政策减轻企业负担，减免退税19.5亿元。第三产业繁荣发展。文化旅游持续升温，举办全国徒步大会等系列活动，投资6000余万元完善了龙江三峡等景区基础设施，全年接待旅游人数、收入分别增长28%、20%。萝北县被列为“2018年中国最美县域榜单”之首，绥滨县荣获“中国最美绿色生态旅游名县”美誉。商贸流通快速增长，南翔海鲜农副产品市场投入使用，比优特时代广场、比优特连锁超市及宏益家电被列为全省实体零售创新转型试点企业。对外合作实现新突破，举办和参加了一系列中俄友好活动，新引进和培育15户外经贸主体，俄罗斯生活体验馆建成开馆。国内合作逐步深化，与汕头初步建立合作机制，开展了“汕头鹤岗年货节”等系列商贸活动。青岛鹤岗商会挂牌成立。

——过去的一年，我们全力激发社会发展的新活力，打基础、利长远，产业支撑能力不断增强。围绕“产业强市”战略，全年推进省、市重点产业项目及转型示范项目78项，预计完成投资31亿元，推动产业项目建设由数量向质量转变。绿色矿业出现新亮点，中海油华鹤增值尿素项目即将试生产，国信通动力电池和智能手机项目首批设备正在安装。帝源矿业选矿生产线投产运行，奥星石墨可膨胀车间主体建成。绿色食品产业整体回升，新龙跃大米胚芽深加工项目建成投产，可口儿大豆产业园、米家乐绿色方便米饭项目正在加快建设。高新技术产业集约发展，开工建设海达锂离子电池负极、中金宝宜分散式光伏发电、禾和生物质发电等一批新技术产业项目，为产业转型注入了新的支撑。全力推进产业园区建设，编制完成经济开发区总体规划、煤化工园区产业发展规划。推动实施精准招商，集中开展外出招商和项目洽谈活动10余次，签订招商项目52个，实际利用省外资金增幅列全省首位。推动创业创新氛围形成，成立了创业创新和电子商务服务中心，培育青年创业标兵、职工“双创之星”、科技创新群体共178个，新增了一批市场主体。推动新技术、新业态、新商业模式广泛应用，在全省率先开通“科淘网”，专利申请量达到215件，促成华升石墨与英国曼彻斯特大学组建实验室。积极发展电子商务，全市建成电商产业园6个、入驻企业105家，六公里无人便利店成为全省首家智能零售企业。培育依靠新业态增收新型农户201户、新型农民创业合作群体9个，建设“互联网+农业”示范基地53个，农产品线上交易额达到8430.7万元。

——过去的一年，我们努力提升城市建设的新品质，强投入、优管理，城乡面貌焕然一新。出台了《鹤岗市城乡规划条例》，全面启动“城乡建设品质提升年”活动，不断丰富和完善城市功能。重大基础设施建设强力推进。全市百万人民期盼多年的鹤佳铁路改造工程正式启动，复兴桥竣工通车，绥名公路改扩建工程全线复工，国防路改扩建、铁西路改建工程建成通车。鹤岗民军合用机场立项审批程序启动。城市人居环境得到改善。开展国家级森林城市创建工作，全年完成造林4.1万亩，栽植树木105万株、义务植树70.4万株，建成国家级绿色矿山5个。建设了一批“口袋花园”“口袋广场”和“口袋绿地”，精心打造了城市出口和中心城区绿化景观，绿色家园的梦想正在变成现实。城市功能不断完善。华能集中供热工程创严寒地区长距离供热首例，新增供热能力1000万平方米，改造老旧供热管网32.9公里。开工建设北山净水厂，改造供水管网、排水管线29.7公里。全年开工建设兴山路等8条城市道路，建成8处停车场，新改建水冲公厕31座。改造老旧楼房249栋，完成绿化修复等配套工程55万平方米。美丽乡村建设全面铺开，对7个城郊村屯实施规划改造，完成投入1700万元。大力实施“厕所革命”，改建农村室内厕所1841座。绥滨县被评定为全省美丽乡村建设三年行动先进县。

——过去的一年，我们切实打造民生保障的新体系，托底线、解民盼，幸福指数稳步上升。推进安居保障惠民，全年各类保障房结转续建复工10346套，回迁安置16830户，着力解决了一大批未回迁安置的历史难题。妥善解决历史遗留的房地产登记问题，办理不动产登记5.4万户，不动产登记中心在全省综合排名第一、入选“全国百佳不动产登记便民利民示范窗口”。推进社会保障惠民，在全省率先完成城乡居民医保制度整合和城乡居民医保手机缴费，新增社会保险参险人员1.7万人。提高城乡低保标准，上调社区干部工资，为重度和困难残疾人发放了护理补贴和救助补贴。推进教育惠民，打造高品质特色幼儿园3所，开办“知行中学”和“知行小学”，改扩建和维修学校35所，鹤岗师专“专升本”考取率超出全省平均水平。推进文化惠民，新建一批乡镇综合文化站、农家书屋和村文化广场，创作各类文艺作品1242件，完成博物馆布展，升级改造图书馆，城乡文化生活品质不断提升。推进健康惠民，国医馆建成使用，20名医生被评为“龙江名医”。萝北县公立医院综合改革连续四年获得全省第一，在全国公立医院改革座谈会上介绍经验，作为我省唯一的县级公立医院被纳入全国现代化医院管理制度试点医院。推进体育惠民，完成体育馆升级改造，成功举办全国大学生武术散打锦标赛等大型赛事活动10余次，家乡运动员刘佳宇获得第23届平昌冬奥会单板滑雪女子U型场地比赛亚军，实现了我国该项目奥运奖牌零的突破，我市在全省第十四届运动会上获金牌67枚，其中雪上项目金牌列全省首位。推进平安惠民，加大信访诉求解决力度，中央第六巡视组向我市移交的145件信访事项全部按期办结或初核，进京越级访、到省越级访分别下降63.7%、13.7%。社会治安体系不断筑牢，深入推进“扫黑除恶”专项斗争，市区实现现行命案零发案，警均打掉黑恶团伙数、破获案件数和抓获违法犯罪人员数排名全省第一，涌现出公安部二级英模李秋元、全省十佳爱民模范张长亮等一批先进典型。全市安全生产形势持续好转，全面完成2018年地方煤矿整治整合任务，地方煤矿连续三年安全生产，煤矿安全监管机制和措施在全省推广。

——过去的一年，我们逐步打造政府工作的好作风，强督查、求实效，营商环境不断优化改善。持续推进简政放权，加强权责清单动态调整，取消权力事项15项，78项社区证明事项全部取消。引进第三方评估机构对全市营商环境进行评价。逐步推开政务服务体系建设，进驻人民办事中心的27家单位窗口全部实行了“一窗受理”服务模式。在全省率先开办政银便民通。持续推进依法行政，组建了市政府法律顾问团，政府部门法律顾问实现全覆盖。全面清理市级行政规范性文件，推进“双随机一公开”监管工作向纵深开展。主动接受人大和政协监督，办理人大代表建议121件、政协委员提案169件。强化行政监察和审计监督，加大领导干部经济责任审计力度。扎实推进政务公开工作，向基层标准化、规范化不断延伸。开通了“民情网”受理群众焦点问题，进一步架起群众与政府沟通的桥梁。

此外，国防动员、双拥工作、统计监测、民族宗教、妇女儿童、外事侨务、防灾减灾、人民防空、地震气象、档案史志、老龄和残疾人事业等工作都取得了新成绩。

各位代表！上述成绩的取得，是省委、省政府和市委正确领导的结果，是市人大、市政协监督支持的结果，是区域各单位和全市人民共同奋斗的结果。在此，我代表市政府，向全市各族人民、各民主党派、工商联、各人民团体和各界人士，向中省直各单位、驻军官兵和所有支持帮助鹤岗发展的各级领导、各方友人表示衷心的感谢和崇高的敬意！

在总结成绩的同时，我们也要清醒地看到，我市在经济社会发展中还存在诸多困难和矛盾：一是经济总量小，煤炭产业独大的问题仍然突出，转型支柱产业尚未形成。二是受市场变化及资金、资源等制约，部分项目进展缓慢，短期内难以形成财力，民营企业经营困难，地方煤矿整治整合给经济增长和职工生活带来影响。三是新增税源有限，而城市建设、社会事业和民生保障等方面刚性支出不断增加，地方性政府债务处于还债高峰期，化解压力增大，财政收支矛盾突出。四是安全生产、社会稳定隐患较多。这些问题，必须着力解决。

二、关于2019年工作

2019年，是新中国成立70周年、全面建成小康社会的关键之年，也是应对诸多困难和挑战、抢抓改革发展重大机遇的攻坚之年，更是我市落实高质量发展要求、加快城市转型的发力之年，做好今年工作至关重要。

按照市委的战略部署，今年政府工作的总体要求是：以习近平新时代中国特色社会主义思想为指导，全面贯彻落实党的十九大和中央、省委经济工作会议精神，聚焦习近平总书记在深入推进东北振兴座谈会上的重要讲话和考察黑龙江的重要指示精神，紧紧围绕实施“四大战略”，发展“五大产业”，打造“三地一区”，建设“一市一乡一胜地”的战略目标任务，进一步解放思想、开拓创新，坚持稳中求进工作总基调，坚持新发展理念，坚持推进高质量发展，坚定不移深化供给侧结构性改革，坚定不移打好“三大攻坚战”，坚定不移重塑投资营商新环境，壮大转型发展新动能，充分释放优势潜能，大力激发内生动力，统筹推进稳增长、促改革、调结构、惠民生、防风险，全面加强党对经济工作的领导，推动经济社会平稳健康发展。

综合考虑各方面实际情况，今年发展的主要预期目标是：地区生产总值增长6%；规模以上工业增加值增长6%；固定资产投资增长6%；公共财政收入增长8%；社会消费品零售总额增长7%；外贸进出口总值增长10%；城镇常住居民可支配收入增长6.5%；农村常住居民可支配收入增长7%。

根据上述总体要求和目标，重点做好以下工作：

（一）扎实推进乡村振兴战略。以农业全面升级、农村全面进步、农民全面发展为目标，优先解决“三农”问题。

打赢脱贫攻坚。按照打赢脱贫攻坚战三年行动的总体部署，实现绥滨县脱贫摘帽，全面做好各项工作的巩固提升。突出抓好产业的可持续、各项扶贫政策的完善和基础设施建设，不断提升脱贫质量，巩固已出列贫困村和已脱贫贫困户的脱贫成效，让贫困户真脱贫、不返贫。

让农业更兴旺。完善农村土地确权工作，不断提高种植业的质量和效益，向观光农业、设施农业、精深加工业发展，把鹤岗好产品卖出高价格。加强与汕头合作，加大农特产品物流支持，降低运输、仓储、时间、运营成本。

让农村更宜居。加快推进美丽宜居村庄建设，打造50个乡村振兴战略样板村。全面清理“大棚房”。扎实推进农村人居环境整治三年行动，推行市场化乡村污水垃圾治理，将秸秆压块与“厕所革命”、可降解垃圾处理、畜禽养殖废弃物利用相统筹，让乡村更干净、更整洁。

让农民多受益。通过村规民约加强村居治理，推动移风易俗，大力培育文明乡风、良好家风、淳朴民风。加强农技推广工作，完善益农信息社建设，让农民更广泛受益。加大金融服务力度，撬动金融和社会资本更多投向农业农村。培养驻村干部、大学生村官、“三支一扶”、大学生志愿者队伍，造就一支懂农业、爱农村、爱农民的“三农”工作队伍。

（二）培育壮大转型发展新动能。重点围绕石墨新材料、绿色农林食品深加工和新能源产业，培育壮大非煤产业，做实做强做优实体经济，真正为城市转型积蓄后劲，尽快形成多点支撑、多业并举、多元发展的转型产业发展格局。

培育壮大石墨新材料产业。对接央企，重点与中国五矿建立战略合作伙伴关系，联合打造石墨资源整合平台，建设集技术研发、成果转化、检验检测、线上线下交易于一体的石墨公共服务创新平台，发展高纯石墨、超硬材料、锂电池、储能电池、石墨烯应用等，创建国内一流石墨企业。加强与全国石墨行业有影响力的民营企业合作，重点引进贝特瑞等行业龙头企业。推动国信通、海达负极、华升石墨、奥星新材料、帝源矿业等一批石墨深加工及应用项目达产达效。做好与清华大学、苏非院等院校的科研合作，切实发挥鹤岗石墨工程中心作用。办好每年一次的石墨产业发展论坛，扩大影响力，力争把鹤岗打造成为全国知名的石墨新材料产业基地。

培育壮大绿色农林食品深加工产业。发挥寒地黑土、非转基因、绿色有机优势，以“粮头食尾”“农头工尾”为引领，加大可口儿大豆产业园项目推进力度，在试生产的基础上，再建设23条生产线。力促东方希望300万头生猪产业项目4个基地投产。突出抓好龙江酒业、新北国啤酒等老字号企业的提档升级，重点推进金鹤精酿啤酒、米家乐绿色方便米饭、绥滨肉鹅产业化等项目。全力打造绥滨优质水稻主产区、萝北杂粮杂豆特色区、市郊蔬菜基地保护区。建设农业物联网试验示范基地，加快稻米加工企业整合与升级改造。大力发展林业经济，成立林业产业发展有限责任公司，扩大林下经济规模，推进森林碳汇项目。推动“韩国商品城”和韩国食品深加工园区建设。整合区域资源，发挥优势企业作用，全面加强与农垦、森工在农业产业化方面的合作，互补互惠互利，推动农林食品产业提质升级。发挥中粮集团在我市组建的股份制粮食发展公司的平台作用，提高农业产业化水平，促进地方农林产品销售。力争用两年时间，把我市绿色农林食品深加工产业打造成支柱产业。

培育壮大新能源产业。全力争创国家级光伏发电领跑基地，带动光伏发电高新技术产业聚集。利用采煤沉陷区土地，建设新能源产业园，发展“光伏+食用菌”“光伏+北药种植”“光伏+特色农业”“光伏+旅游”等模式，有效推动一二三产融合，丰富产业布局，形成规模集聚效应。推进垃圾发电、禾和生物质发电、鑫塔水泥余热发电等项目。做好《鹤岗市分散式风力发电规划》与省整体规划的衔接工作，争取更多风力发电项目获得政策支持。力争在培育壮大新能源产业方面，为我省煤城转型作出示范带头作用。

（三）促进现有企业优质高效发展。统筹处理好稳增长和调结构、扩需求和促转型、抓改革和防风险、促发展和惠民生、谋长远和抓即期之间的关系，抓好存量经济运行，在优化产业结构、提升质量效益上做实工作。

重视支持现有企业高效发展。推动中海油华鹤新华煤矿项目建设，争取早日落地达效；推进华能鹤电利用人才、技术、装备和管理的优势，深化电量营销攻关、煤炭保量控价攻关和成本管控攻关；支持龙煤鹤矿加快鸟山矿井建设，盘活现有资产，让老企业焕发新生；支持农垦、森工等所属企业发展；支持国信通动力电池达产达效；支持征楠煤化工扩大有效投资，建设银鹤纸业，延伸产业链条。强化规上企业培育，稳住现有，培育更多。落实帮扶亏损困难企业专项方案，强力推进规上企业扭亏工作，努力降低亏损面。加强煤、电、油、气、运等生产要素协调，帮助企业扩大产销、降低生产成本。建立完善与现有企业定期联系、运行分析调度等制度，提供精准服务，促进问题解决，当好企业的服务员。

重视支持地方煤矿转型发展。必须认识到，如果还是煤字当头，城市必然会衰落，必须增强转型发展的危机感、紧迫感。同时也必须看到一个时期还脱离不了煤经济的支撑，在大力发展非煤产业的同时，还要坚持稳定煤炭生产，提高煤炭产业的综合效益，为转型发展积累财富赢得时间。要大力抓好煤矿整治整合，培育优质产能，促进煤炭产业安全健康发展。要大力支持地方煤矿转型发展，特别要给予更加优惠的政策，支持退出企业新上非煤项目。

提升企业科技创新能力。推进新一轮科技型企业三年行动计划，以企业需求为导向，深入挖掘技术需求，带着需求寻找研发团队，合作研发，精准对接，成果就地转化。与科研院所、大专院校合作，完成2户企业科学家工作室的组建工作，加速科技成果落地生效。推进科技计划项目备案工作，鼓励企业技术创新，加强科技培训和政策支持，不断提升企业创新能力和市场竞争力。

加强人才储备。实施好“头雁”行动，筑巢引凤留凤，探索实施领军人才“一卡通”制度，对高端人才在解决配偶工作、户口、社会保险、医疗保险及子女入学等方面出台支持措施、提供帮办服务，吸引人才、留住人才、用好人才，为企业优质高效发展提供人才保障。

（四）大力发展新经济、新业态。结合实际，深入挖掘优势资源，用新业态新模式推动自身优势潜能转化为经济优势，为转型发展增添新动力。

抓好旅游康养产业迅速升级。推动生态优势向产业优势转化。打造鹤岗特色旅游品牌，主动融入全省旅游康养产业布局，编制《鹤岗市全域旅游总体规划》，支持两县、宝局、森工区域旅游发展。推进旅游供给侧改革，积极培育文化、体育、旅游相融合的旅游产品，努力打造精品旅游线路，持续叫响“游龙江三峡，赏两国风光”旅游品牌。抓好旅游基础设施建设，支持社会资本进入旅游康养产业，积极引入龙头企业，带动产业迅速升级。

推进商贸物流产业快速增长。加快推进泓博来农副产品交易市场、南翔物流二期等商贸市场建设。谋划推进潮汕产品大市场建设。加强外经贸主体的培育和服务力度，确保五矿萝北公司健康稳定发展。组建鹤岗市对外经济技术合作有限公司，鼓励企业在境外设立加工厂、经营展示中心和零售商店，推动我市商品走出国门。进一步完善“好品鹤岗”电子商务平台服务体系建设，引进第三方知名电商。深化与哈工大机器人集团战略合作，推进智慧城市建设，发展新一代信息技术产业。

推进矿产资源转化成产业。巩固并延伸绿色矿业产业链条。创新矿产资源找矿开发新模式，利用安泰能源开发公司平台，继续推进城市地质调查，加大地热、浅层地温能、矿泉水等矿产资源勘查开发力度，全面摸清耕地、草原、河流、林业、湿地等自然资源“家底”。严格规划管控，优化开发布局，合理配置资源，规范生产秩序，推动砂石土矿产资源有序开发和合理利用，尽快促进资源转化成产业。

（五）增强发展内生动力。加快发展是解决当前困难的唯一途径；解决发展的短板问题关键是激发内生动力，我们必须主动作为，主动出击。

掀起项目建设新高潮。牢固树立抓项目就是抓发展、抓后劲的理念，把扩大有效投资作为首要任务，开展项目建设攻坚。要高质量谋划储备项目，紧盯东北振兴、煤城转型等政策要点，从政策导向衍生项目，从转型方向规划项目，与科研机构合作开发项目。力求有目标、有任务、列清单，精心谋划一批优质项目。要狠抓项目推进，进一步健全完善项目并联审批各项制度，重新修订公布项目审批流程，全面落实“一会审定”“三方承诺”“公开公示”“一报三制”“督办问责”项目建设快速反应机制，坚决处理项目推进中的梗阻行为，切实提高服务项目建设的质量和效率。要主动精准务实招商，重点实施产业招商、以商招商、全员招商，要领导带头，担任务，扛指标，身体力行、脚踏实地，主动谋划、宣传联络，洽谈推动、解决难题，增强招商引资针对性，提高成功率，促进项目生成。要严格考评，增强考评的真实性、针对性和实效性，完善激励奖惩机制，奖优罚劣，尽快形成人人关注项目建设、支持招商引资的良好氛围。

加强园区载体建设。完成《石墨园区产业发展规划》编制，启动《绿色食品产业发展规划》编制，完善省级经济开发区功能，组建经开区配售电公司，降低企业用电成本。针对影响民营经济、中小微企业发展的用地、规划、环评、建设等方面的突出问题，新建2个中小微企业园区，为中小微企业发展提供要素服务支撑。加快推进路、水、电、热等基础设施建设，抓好土地收储，做好土地预留，整合棚改腾空土地，提升项目承载能力。

大力优化营商环境。坚持“营商环境只有更好，没有最好”的原则，以“办事不求人”为目标，着力打造高效透明的政务环境、公平正义的法治环境、诚信守约的人文环境以及风清气正的政治生态环境，提高各类市场主体对营商环境的满意度。深化“放管服”改革，继续简政放权，通过改革放出活力、管出公平、服出效益。加强“智慧政务”建设，推进“互联网+”政务服务“一门、一网、一窗”建设。推动行政审批事项和公共服务事项“最多跑一次”改革。

加快民营经济发展。民营经济弱是我们的短板，也是经济发展的潜力所在。要制定并落实支持民营经济发展若干优惠政策，围绕产业项目建设、促进创业创新、金融支持实体经济、人才保障等方面打造“政策洼地”。新上工业企业项目的环评等14项中介服务费由政府购买。全面推进企业投资项目网上备案管理，建立健全容缺审批、委托审批等机制，按照“非禁即入”原则，实行“负面清单”管理机制。不折不扣落实税收优惠政策，确保民营企业应享尽享。支持企业打造优势品牌，对新获得国家级标识品牌的企业给政策支持。加大金融服务支撑，合理运用担保平台撬动银行机构资金流向民营经济领域。要坚决做到“企业有困难必帮解、有要求必回应”，把为企业解决问题的过程作为倒逼做好服务的过程。切实保护民营企业家的人身和财产安全，在全社会形成尊商富商重商亲商的良好氛围，让企业家安心放心。提高民营企业发展规模和质量，加快形成更多新的经济增长点。

深化重点领域改革。坚持“巩固、增强、提升、畅通”的“八字方针”，推动供给侧结构性改革，增强应对不确定性的能力，增强经济发展韧劲。支持农垦、森工和龙煤改革，做好接收企业办社会职能和行政移交工作。针对效率低下、管理涣散、缺乏竞争力和创造力的问题，推动经营性事业单位企业化改革，探索引进社会资本，促进混合所有制改革。以产业发展引领公司化改革，完成文旅、粮食、建设、交通集团组建，支持广电传媒集团、林业集团继续深化改革。充分释放改革带来的新动力，切实以改革新突破打造转型发展新优势。

强化区域合作。打破以往在产业项目建设、重大基础设施建设等方面存在的藩篱制约，促进区域内发展要素优化组合，推动区域协调发展。力求鹤矿、宝局、鹤北与市政的融合有实质性的突破，推动县区、中省直驻鹤单位、大型企业融合到发展中来，互相借助优势，释放潜力，构建城乡协同发展、区域板块一体化发展新格局。强化城市对内对外的辐射功能，让区域合作成为我市经济发展新的增长极。推进军民融合深度发展。深化与汕头对口合作。

（六）完善城市发展功能。要抢抓国家加大基础设施等领域补短板力度的重要窗口期，取长补短，提升城市品位，打造和谐优美的宜居环境。

重大基础设施建设要有新突破。加快推进鹤佳铁路改造工程，全面完成域内征地拆迁、“四电”迁改及道路改移等相关工作，3月份全线开工建设，力争早日建成通车，让鹤岗百姓在家门口乘车直接进入全省高铁网；推动伊春—鹤岗客专深度谋划，加快进程；全力推动支线机场前期工作，争取有实质性突破；加快推进关门嘴子水库、细鳞河水库除险加固等水利工程前期工作，争取早日开工建设；重点完成绥名公路、小鹤立河危桥改造及鹤伊公路鹤岗出口段大修工程，有效推动重点交通基础设施建设。

城市路网通达性要有新提高。完善城市交通框架体系，继续推进“一环五纵七横”城市道路工程建设，进一步解决破损路、断头路、瓶颈路等影响群众生产生活问题。升级改造财富路、南选路等8条城区道路，确保“经一纬五”路实现通车，贯通西部环线，确保棚改项目小区外道路全线开工建设，让百姓出行不再受堵。

暖屋子、放心水要有新保障。全力推进华能集中供热后续项目建设，淘汰城区所有小锅炉，并入集中供热网，实现城区集中供热全覆盖，改造“跑冒滴漏”老旧供热管网14.4公里，让所有的屋子都暖起来，确保百姓供暖期不受冻。继续实施供水老化管网和水表出户改造工程。加快推进北山净水厂新建工程，大幅度增加供水能力，力争用2年时间建成一座现代化水厂，尽快让百姓喝上放心水。同时，全力推进主城区燃气管线建设，扩大清洁能源覆盖面，新增燃气用户1.5万户，让更多的百姓用上清洁能源。

支持六区特色发展。准确把握六区核心优势，定位区域功能，最大程度凸显特色。支持东山区腾空土地清退修复，再塑工业强区辉煌；支持兴安区完善基础设施，让兴安百姓衣食住行更加舒适；支持向阳区提升区域品质，先行建成森林街区、文明街区；支持工农区提升服务功能，让商贸中心更加繁华；支持南山区按照主城区标准加快建设，逐步实现由矿区向主城区转变；支持兴山区打造“飞机房”历史文化街区，使老城区焕发新生机。

城市管理水平要有新提升。对市区主次街道实施分类管理，集中整治违法建设、违规占道经营等行为，营造整洁、美观、舒适、有序的市容市貌。新建一批小型停车场和停车位，缓解城区“停车难”的问题。积极推广新能源汽车使用，合理规划建设天然气加气站和电动汽车充电桩。优化城乡公交线路，引导市民错时乘车，提升百姓出行舒适性。抓好新建道路路灯亮化，改造提升老旧路灯，健全背街背巷路灯设施。配齐新型环卫设施，新建移动式垃圾转运站和水冲式公厕，多点布局道路、园林、广场绿地，对财富路、兴山路、南奋斗路等重要节点进行绿化生态修复。规划建设麓林山风景区，完成欣虹湖湿地公园建设。推进城市管理综合执法改革，提倡“温情”的文明执法方式，创建共谋、共建、共治、共享的城市管理环境，优化城市管理和服务，改善市民生活质量。

（七）用绿色发展理念推进生态治理。生态环境是我们的优势，又是我们当前的痛点，城区的山体、河流、土地、湿地损毁严重。必须下决心花大力气，持之以恒修复治理，让我们的家乡天更蓝、山更绿、水更清、环境更优美，生活更舒适。

高标准推进森林城市创建，让我们的城市绿意盎然。以突出森林主题为标志，把森林城市创建与城乡建设、生态建设有机融合、相互促进，让广大人民群众享受到生态环境的福利，成为我市经济社会发展的潜力和支撑。坚持规划统领，对照四大体系、五大类46项指标，达到城市公园化、公园景区化的要求。强化森林街道、森林社区、森林乡镇、森林学校、森林企业建设与森林城市创建目标协调推进。紧盯绿化、美化、品质、融合、特色等关键点和薄弱环节，集中打好歼灭战。要全面动员、全民参与、全社会行动，人人参与到创森工作中来，做到我参与、我奉献、我享受。支持区属公园建设，做到市助区办，重点支持东山石头河沿河公园、南山体育公园、向阳植物园建设。要加大动植物保护力度，全域禁捕，公园禁采，对非法占林、占山、占水、占湿地的行为，联合执法打击。

从根儿上全面治理黑臭水体，让我们的城市依水而秀。全面落实河湖长制。推进水污染防治工程，加快推进园区污水处理厂、西部污水处理二期和绥东镇、忠仁镇污水处理厂建设，推进地表水水源地达标建设。今年是“两河十四沟”黑臭水体治理工作最关键的一年，工程量大，任务繁重，要巩固去年工作成果，强力实施二期工程，通过实施截污、清淤、活水、秀岸、添绿等标本兼治措施，实现老头沟、头道沟、二道沟、前进沟全面竣工，形成带状水系公园。启动西山沟、石头河等9条河流治理工程，2020年全部完成黑臭水体治理，让城区的每一条河沟都达到水清岸秀的目标标准，为全市人民提供优美的生活环境。

解决环保重点难点问题，让我们的城市风清气爽。组织编制城乡垃圾综合治理专项规划，全力推动生活垃圾焚烧发电和餐厨垃圾无害化处理项目建设，补齐垃圾污染治理短板。持续推动大气污染防治工程，启动实施市建成区内35吨以下、两县建成区10吨及以下燃煤锅炉淘汰计划。严控秸秆焚烧，着力提高重污染天气研判和处置能力。启动机动车尾气监管平台，全年PM2.5稳定达到国家大气环境二级标准以上。以“山水林田湖草”项目为契机加大矿山地质环境治理力度，对郊区非煤矿山进行生态修复和综合利用。继续对违法用地问题进行严厉打击整治，实现基本“清零”，规范土地管理秩序。启动林区土地清理。推进轮作休耕，加大黑土地保护力度。加大地下水压采工作力度。

（八）推进民生热点难点问题解决。更加关注补齐民生领域短板，让人民群众共享改革开放、东北振兴、鹤岗转型发展成果。

抓就业创业，稳民生之本。坚持就业是民生之本、创业是就业之源的理念，统筹抓好煤矿下岗职工、农村转移劳动力、城镇困难人员、退役军人等各类群体就业。落实公益性岗位补贴、灵活就业人员补贴等各项就业优惠政策，为符合条件的中小微企业提供“援企稳岗”服务，做好龙煤转岗分流安置人员待遇发放工作。建立求职人员和用工需求数据库，把劳动力市场搬到社区、村屯，把岗位送到百姓家门口。扩大就业见习补贴人员范围、提高补贴标准。落实好创业担保贷款政策，设立300万元大学生创业种子资金。对首次创办小微企业或从事个体经营的重点群体给予一次性创业补贴，对农民创业主体吸纳贫困家庭劳动力稳定就业1年以上的给予一次性奖励补贴；加快建设重点群体创业孵化载体，提高被评为国家及省级创业孵化基地的奖补标准。

抓社会保障，保民生之需。加强“五险合一”社会保险信息化建设，完善城乡居民基本医疗保险运行机制，实现基本养老、基本医疗、失业、工伤、生育等保险法定人员全覆盖。完善民政基础设施，加快推进东郊殡仪馆二期、民政局福利院改造工程建设。落实农民工工资支付保障制度，开展治欠保支专项行动。认真落实国家政策，切实做好退役军人安置和优抚工作。对关停煤矿的下岗职工实行特殊群体最低保障制度。持续推进保障性安居工程，结转续建工程复工建设13226套，完成拆迁1万户、实现安置1万户，让更多的老百姓迁回新居。加大公共租赁房建设力度，发放租赁补贴7500户，解决城镇低收入群体住房困难。扎实推进各类社会保险统筹扩面，实行无类别化的“综合柜员制”“互联网+社保”，解决社保办事流程不优、群众排长队的问题。

解决历史遗留问题，解民生之痛。妥善处置“烂尾楼”、百姓回迁和“弃管楼”、物业管理服务不到位的问题，随时排查、随时整治、随时化解。组织推进老旧小区改造，争取列入国家试点城市，完成28万平方米改造后小区的绿化硬化工作。坚定不移地全面完成不动产权证办理工作。简化房屋专项维修基金程序，便民利民惠民。

（九）大力推动社会事业发展。紧紧围绕全市人民最关心最直接最现实的利益问题，努力让鹤岗百姓拥有更多彩的文化、更好的教育、更高水平的医疗服务和更广泛的体育活动。

坚持教育优先。持续改善办学条件，完成煤城小学综合楼、蔬园小学食宿楼和第二十二中学食宿楼、风雨操场建设，启动第十七中学建设工作。推进体育中考改革，引领学生重视体育锻炼，提升身体素质。实施“护眼工程”，推进校园照明设备改造。全面推进“智慧教育”工程。在义务教育“基本均衡”的基础上着力解决“择校热”“大班额”等突出问题，不断向“优质均衡”迈进。加强教师队伍建设，招录各类紧缺中小学教师150名。聚焦师德师风建设，严惩“收红包”“拉赞助”行为。打造学生欢迎、家长放心、教师满意、社会认可的教育。

坚持卫生惠民。深化医药卫生体制改革，加速健康服务产业融合发展。完善分级诊疗制度，提升基层医疗机构门诊质量。创新互联网医疗服务应用。引导吸纳社会资本，推进医养康护结合模式构建。落实卫生惠民举措，启动“优质服务基层行”，推进14类56项基本公共卫生服务，开展农村贫困人口18种大病专项救治，为贫困的肝炎、肺结核、白内障患者提供免费治疗。全面启动城镇职工大病保险工作，将医疗保障年度累计支付限额由20万元提高到40万元，将门诊普通慢性疾病病种增加到20个病种，将40种谈判药品和17种抗癌药物纳入医疗保障范围，减轻患者负担。创新互联网医疗服务应用，推广智慧医疗，实现基本医疗保险、大病保险和医疗救助的“一站式”结算，实现省内异地个人账户划卡直接结算，实现“互联网+医保”全覆盖。严厉打击医疗保障领域欺诈骗保行为。

打造特色文体品牌。培育鹤岗特色文化品牌，引领文化时尚，增强城市的文化内涵。织密全民健身场地设施网络，利用闲置场所创办社区文体活动站，打造文体“15分钟”健身圈。在政策、场所等方面支持群众团体提档升级，发挥作用。完善公共文化体育服务体系建设。创建“全民运动健身模范市”，增添城市活力和影响力。推动“体育+产业”发展，积极培育、开展具有鹤岗特色的群众性体育赛事活动，开展在外鹤岗人回乡过节、避暑休假活动，增强城市向心力。

（十）提高社会治理水平。全面加强安全生产、社会治安、社区治理工作力度，打造共建共治共享的社会治理格局，促进社会和谐稳定、百姓安居乐业。

抓好安全生产工作。突出抓好以煤矿为重点的安全生产工作，提高地方煤矿质量标准化建设，不达标的一律不予验收、不准生产。实施安全生产风险分级管控、隐患排查治理双重预防机制。抓好特种设备、交通运输、非煤矿山、危险化学品、建筑行业、药品等重点领域以及学校、商场等人员密集场所的安全。抓好食品安全工作，实施“食安鹤岗”品牌创建，开展好“放心肉菜示范超市”“食品生产示范企业创建”活动。

抓好社会治安工作。加强立体化、信息化社会治安防控体系建设，构建纵向到底、横向到边的公共安全网络，提高人民群众的安全感。加强公安战线基层基础工作，建好公安综合训练中心，锻造公安铁军。纵深推进“扫黑除恶”专项斗争，坚决做到斩草除根，除恶必尽。保持高压态势，对各种违法犯罪坚决实行严防、严打、严惩，把鹤岗打造成平安清净之地。

抓好社区治理工作。落实好社区减负各项政策，完善抓落实的工作机制和办法，推动社区治理创新落地见效。打造“智慧民政”，启动运营12349公益服务热线，面向全市社区居民提供基本公共服务。推动“阳光工程”建设，在六区各打造一个“阳光社区服务中心”，实施“十百千工程”，组织引导十个社会组织、百名爱心人士和千名志愿者投入社区服务。积极争取国家第四批居家养老服务试点城市，加强养老机构监管，提升服务质量。成立鹤岗市社会组织发展服务中心，参与社会治理和对外交流。启动“文明祭祀”工程，引导群众移风易俗。全面提升社区居民参与能力、依法治理能力、智能化应用能力。全面提升社区服务供给能力，不断改善社区人居环境。

各位代表，习近平总书记告诉我们“幸福都是奋斗出来的”！诸多重担已经摆在我们每个人的面前，政府系统不能有丝毫的犹豫和懈怠，要迎着困难上、顶着风雨走，展现新形象，再有新作为。要解放思想，树立强烈的改革意识、创新意识和市场意识，积极探索新思路、新办法、新举措，主动适应新时代、应对新挑战、推动新发展。要强化履职尽责，大力倡导敢闯敢试、敢于碰硬、敢于承重的风气，强化责任意识、担当意识，对看准的事情、定下的目标一抓到底，推动各项工作干在实处、走在前列。要坚决反对形式主义和官僚主义，时刻把群众安危冷暖放在心中，察实情、出实招、办实事、求实效。要提高政治站位，严格落实全面从严治党的各项纪律要求，落实“一岗双责”，严格监督检查，加大行政监察和财政、审计监督力度，树立过紧日子、苦日子的思想，进一步从严控制一般性支出，营造良好的干事环境，打造风清气正的廉洁政府。

新起点孕育新希望，新希望催生新动力。只要我们紧紧扭住高质量发展不放松，更好地认识和解决当前问题，更好地精准有效施策，更好地践行以人民为中心的发展思想，敢于追梦，努力奔跑，一起拼搏、一起奋斗，就一定能把鹤岗的事情办好，就一定能让鹤岗人民过上更加幸福美好的生活，就一定能创造经得起历史检验、人民满意的新业绩！
